# A phase 1 study comparing the proposed biosimilar BS‐503a with bevacizumab in healthy male volunteers

**DOI:** 10.1002/prp2.286

**Published:** 2017-02-20

**Authors:** Naoyuki Tajima, Alberto Martinez, Fumiaki Kobayashi, Ling He, Peter Dewland

**Affiliations:** ^1^ Daiichi Sankyo Co., Ltd.1‐2‐58 HiromachiShinagawa‐kuTokyo140‐8710Japan; ^2^ Daiichi Sankyo Development Ltd.Chiltern Place, Chalfont ParkGerrards CrossSL9 0BGUnited Kingdom; ^3^ Daiichi Sankyo Pharma Development399 Thornall StreetEdisonNew Jersey08837

**Keywords:** bevacizumab, biosimilar, BS‐503a, healthy male volunteers, pharmacokinetics, safety, similarity

## Abstract

This is a randomized, double‐blind, single‐dose, parallel group phase 1 study to assess pharmacokinetic similarity, safety, and tolerability of BS‐503a, a proposed bevacizumab biosimilar. A total of 114 male healthy subjects were randomized (1:1) to receive a single 3 mg/kg intravenous dose of either BS‐503a or bevacizumab (Avastin^®^). Pharmacokinetic (PK) blood samples were collected up to Day 78, and serum drug concentrations were measured using a validated enzyme‐linked immunosorbent assay. Pharmacokinetic similarity was evaluated using area under the serum concentration‐time curve from zero to infinity (AUC_inf_) as a primary PK parameter, and maximum serum concentration (*C*_max_) and area under the serum concentration‐time curve from zero to the last measurable time (AUC_last_) as secondary PK parameters. The 90% confidence intervals (CIs) of geometric mean ratio of AUC_inf_ ranged 0.980–1.105, which met the predefined criteria of 0.80–1.25. The 90% CIs of geometric mean ratios for *C*_max_ and AUC_last_ were 1.009–1.125 and 0.982–1.096, respectively, falling into the same criteria. At least one drug‐related treatment emergent adverse event occurred in 18 and 21 subjects treated with BS‐503a and bevacizumab, respectively. The most common adverse events were headache, epistaxis, and rhinorrhea. Most adverse events were mild or moderate; however, one drug‐related serious adverse event of duodenal ulcer perforation was reported by a subject 47 days after treatment of BS‐503a. In conclusion, BS‐503a was demonstrated to have highly similar PK to bevacizumab and adverse events observed were consistent with those observed for bevacizumab.

## Introduction

Bevacizumab (Avastin^®^, Genentech, South San Francisco, CA) is a recombinant humanized monoclonal antibody directed against vascular endothelial growth factor receptor (VEGF). It recognizes and neutralizes all isoforms of human VEGF and blocks its signal transduction. This ultimately decreases the number of endothelial cells and the quantity of microcapillaries in the tumor tissue. Bevacizumab can also lead to a reduction in the vascular permeability and thus contribute to the inhibition of tumor progression (Ferrara et al. 2003, 2004; Jain 2005). Bevacizumab combined with other neoplastic agents has been approved for the treatment of advanced non‐small‐cell lung cancer (NSCLC), breast cancer, colorectal cancer, renal cell carcinoma, ovarian cancer, and malignant glioma (Food and Drug Administration, 2013; EMA, 2014).

A biosimilar is a biologic medicinal product not identical but similar to the originator. Biosimilarity is evaluated based on totality of the evidence taking a stepwise approach to demonstrate similarity. Regulatory guidelines suggest focusing on the quality attributes of the products (both innovator and biosimilar products) as the first step to demonstrate similarity. Once the quality attributes indicate similarity, nonclinical evaluation including in vitro and in vivo studies are the next step in the process. The final step is to conduct clinical studies to complete the evaluation of similarity (Committee for Medicinal Products for Human Use (CHMP), 2014; US Department of Health and Human Services, 2012) which comprise a demonstration of PK bioequivalence and clinical safety.

BS‐503a is being developed as a proposed biosimilar of bevacizumab by Daiichi Sankyo. BS‐503a is a recombinant humanized IgG1 monoclonal antibody which has demonstrated high similarity to bevacizumab in quality attributes including primary structure, higher order structure, biological activity, binding affinity to VEGF, and PK in monkeys. In addition, no new findings were observed for BS‐503 in monkey toxicology studies when compare to bevacizumab.

A human pharmacokinetic (PK) study in healthy subjects, as the next step, was planned to demonstrate similarity. During clinical development, bevacizumab was administered in cancer patients and therefore safety information of bevacizumab in healthy subjects is limited. A study in the literature in which bevacizumab was infused into the brachial artery of 34 healthy male subjects had no side effects resulting from bevacizumab exposure. However, bevacizumab systemic exposure in that study was lower than that achieved during clinical use (Thijs et al. 2013). This study provides some support for the safe administration of bevacizumab in healthy male subjects.

The aim of this study was to demonstrate PK similarity in healthy male subjects and safety of BS‐503a when compared to bevacizumab.

## Materials and Methods

### Study population

A total of 114 male healthy subjects were randomized to one of two treatment groups prior to dosing to receive either a single intravenous (IV) dose (3 mg/kg) of BS‐503a or bevacizumab. Subjects needed to be 18–55 years old, with a body weight between 67 and 100 kg, and a body mass index between 20 and 28 kg/m^2^ at screening. Exclusion criteria included history of gastric or duodenal ulcers, bleeding or surgery or previous exposure to recombinant monoclonal antibodies. Informed consent was obtained from all individual participants included in the study.

### Study design

This was a randomized, double‐blind, single‐dose, parallel group Phase 1 study in healthy male subjects. The primary objective of this study was to demonstrate pharmacokinetic similarity, as assessed principally by the area under the concentration‐time curve from time zero extrapolated to infinity (AUC_inf_) of BS‐503a compared to bevacizumab. Sample size was estimated based on assumption that AUC_inf_ had an intersubject coefficient variation of 30% (Zhi et al. 2011). After subjects were screened to ascertain their eligibility for the study according to the inclusion and exclusion criteria in the protocol, subjects were randomized to one of two treatment groups to receive either a single IV dose (3 mg/kg) of BS‐503a or bevacizumab for 90 min. The subjects were followed up for assessment for 77 days.

### Pharmacokinetic analysis

Pharmacokinetic blood samples were collected at predose, 0.75, (i.e., during infusion) 1.5, (i.e., at end of infusion) followed by 2, 3, 4, 6, 12, 24, and 48 h and Days 4, 5, 6, 7, 10, 14, 21, 28, 42, 56, and 78 (follow‐up visit) after end of infusion, and serum was stored at −70°C until assay. Serum drug concentration was measured using a validated enzyme‐linked immunosorbent assay. Quantification range of the assay was 0.2–100 *μ*g/mL. Precision and accuracy in interassay reproducibility test were <14.9% and −16.0–12.0%, respectively.

Serum concentrations versus time data were analyzed using noncompartmental methods. The following PK parameters were estimated for bevacizumab and BS‐503 treatments: AUC_inf_, maximum concentration (*C*_max_), time of *C*_max_ (*T*_max_), area under the concentration‐time curve from time zero to time of last measurable concentration (AUC_last_), terminal elimination half‐life (*t*½), total body clearance (CL), and volume of distribution (Vd).

A mixed effects model with treatment as a fixed effect was used to compare natural‐logarithmic transformed PK parameters (AUC_inf_, AUC_last_, and *C*_max_) between two treatment groups (BS‐503a vs. Bevacizumab). Geometric mean test/reference ratios and their corresponding 90% confidence interval (CI) were calculated by anti‐logarithmic transformation. Pharmacokinetic similarity was concluded if the 90% CIs were contained within 0.800–1.250 for AUC_inf_.

### Safety evaluation

Safety and tolerability were assessed by evaluation of adverse events (AEs), physical examinations, vital signs, ECGs, and clinical safety laboratory tests. Immunogenicity was assessed through characterization of incidence and titer of anti‐drug antibodies.

### Immunogenicity

Blood samples for immunogenicity assessment were collected at predose, Days 14, 28, 56, and 78 (follow‐up visit) after end of infusion and serum was stored at −70°C until assay. Anti‐drug antibody (ADA) was evaluated using a validated electrochemiluminescent immunoassays (ECLIA) assay. All samples were first analyzed in a screening assay. When result was above the screening cut‐off point, the samples were additionally analyzed in a secondary confirmatory assay (specificity assay). In cases where the assay signal was reduced after the addition of an excess of bevacizumab beyond the validated confirmatory cut‐off point, a sample was reported as positive.

## Results

### Subject disposition

A total of 114 healthy male subjects were randomized. One subject treated with BS‐503a was lost after Day 56, and 113 completed subjects were included in the PK analyses. The baseline demographic characteristics were similar between arms (Table 1).

**Table 1:** Demographics of enrolled subjects

	BS‐503a	Bevacizumab
Subjects	57	57
Age^a^	35.5 ± 9.65	37.7 ± 10.68
Body weight (kg)^a^	80.47 ± 8.171	77.31 ± 6.662
Body mass index (kg/m^2^) a	24.91 ± 2.038	24.95 ± 1.876
Race		
Asian	3	6
Black or African American	5	10
White	46	37
Mixed/Other	3	4
Ethnicity		
Hispanic	3	2
Non‐Hispanic or Latino	54	55

a Mean ± SD.

### Pharmacokinetics

**Figure 1:**
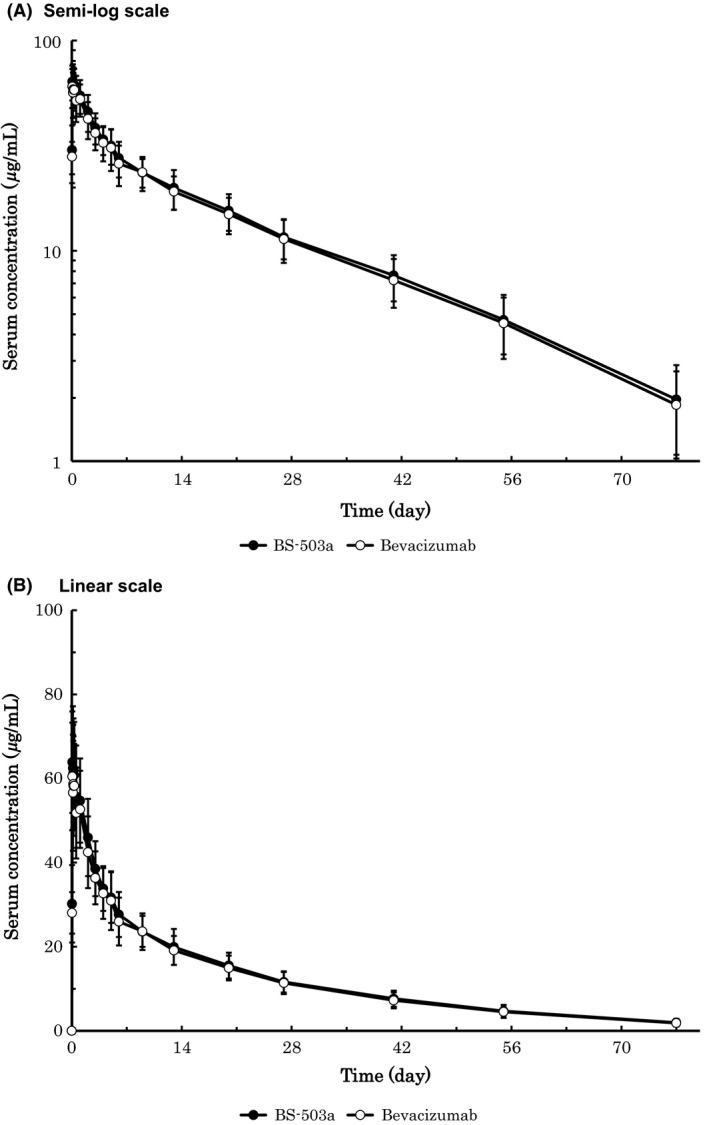
Time‐serum concentration profile of BS‐503a and bevacizumab (mean ± SD).

Time versus serum drug concentration profile and PK parameters are shown in Figure 1 and Table 2, respectively. The curve matched well and no notable differences in PK parameters were observed between two groups.

**Table 2:** Pharmacokinetic parameters of BS‐503a and bevacizumab (mean ± SD)

	BS‐503a	Bevacizumab
Subjects (*n*)	57	56
AUC_inf_ (day × *μ*g/mL)	971.21 ± 186.25	931.38 ± 176.8
AUC_last_ (day × *μ*g/mL)	913.57 ± 161.44	879.13 ± 152.36
*C*_max_ (*μ*g/mL)	70.8 ± 13.56	66.1 ± 10.63
*T*_max_ (h)^a^	1.5−49.35	1.5−25.50
CL (mL/day/kg)	3.21 ± 0.67	3.33 ± 0.61
Vd (mL/kg)	83.18 ± 14.26	85.87 ± 13.31
T½ (h)	441.38 ± 84.85	438.37 ± 81.41

a *T*_max_: minimum − maximum, time after start of infusion.

Statistical analysis of the test product (BS‐503a) and reference product (bevacizumab) is shown in Table 3. Based on the primary variable of AUC_inf_, similarity was confirmed as the test/reference mean ratio was 1.041 and the corresponding 90% CI was 0.980–1.105. The 90% CIs of test/reference mean ratios for *C*_max_ and AUC_last_ were 1.009–1.125 and 0.982–1.096, respectively, falling into the same criteria for bioequivalence.

**Table 3:** Summary of statistical analysis of serum bevacizumab pharmacokinetic parameters

	Geometric mean		Ratio	90% CI
	BS‐503a	Bevacizumab		
AUC_inf_ (day × *μ*g/mL)	952.939	915.738	1.041	0.980, 1.105
AUC_last_ (day × *μ*g/mL)	898.969	866.822	1.037	0.982, 1.096
*C*_max_ (*μ*g/mL)	69.546	65.289	1.065	1.009, 1.125

CI: confidence interval.

### Safety

Summary of safety assessment is shown in Table 4 and 5. A total number of 114 subjects were treated with BS‐503a or bevacizumab. At least one drug‐related treatment‐emergent adverse event (TEAE) occurred in 18 and 21 subjects treated with BS‐503a and bevacizumab, respectively. The common AEs were headache, epistaxis, rhinorrhea, nasopharyngitis, and somnolence, and most were mild or moderate.

A serious AE of duodenal ulcer perforation was reported by a subject 47 days after treatment of BS‐503a and the subject recovered after surgery. The subject had no apparent contributing factors from the medical history but tested positive for *Helicobacter Pylori*, for which he received anti‐helicobacter triple therapy.

A single 3 mg/kg IV dose of BS‐503a or bevacizumab did not have any clinically significant effect on the change from baseline in 12‐lead electrocardiogram measurements, vital signs, or clinical laboratory tests.

### Immunogenicity

All baseline samples for the both treatment groups showed negative. ADA was detected in all samples on Day 14 except for one subject in the bevacizumab‐treated group. Incidence of ADA decreased according to the decline of serum drug concentration. On Day 78, seven and six subjects from the BS‐503a and bevacizumab group, respectively, remained positive.

## Discussion

This is the first study where BS‐503a was administered to healthy human beings to demonstrate similarity of PK profile to the originator bevacizumab. Since bevacizumab is administered intravenously, as guideline suggested, AUC_inf_ was set as primary parameter estimate and AUC_last_ and *C*_max_ as the secondary (Committee for Medicinal Products for Human Use (CHMP), 2014; U.S. Department of Health and Human Services, 2014). As shown in Figure 1 and Table 2, both primary (AUC_inf_) and secondary (AUC_last_ and *C*_max_) parameters met the predefined criteria for bioequivalence, suggesting that PK of BS‐503a is similar to that of bevacizumab.

PK parameters obtained in this study were consistent with previously reported values in cancer patients; however, interindividual variability was small (Zhi et al. 2011). During development of bevacizumab in cancer patients, body weight, gender, serum albumin concentration, and serum alkaline phosphatase were identified to have influence on PK of bevacizumab (Lu et al. 2008). Disease status in a patient can influence PK behavior with, for example, serum albumin and alkaline phosphatase levels being influenced by disease severity and correlate with patient condition. In contrast, in this study, only male subjects with narrow range of body weight and in good health were enrolled which may explain the small variability in PK parameter observed. The advantage of a small variability is that sensitivity to detect PK deference could be improved to allow better evaluation of similarity.

**Table 4:** Summary of treatment‐related adverse events

	BS‐503a	Bevacizumab
Number of subjects	57	57
Total number of TEAEs	73	107
Number of subjects with a TEAE	18	21
Number of subjects with serious TEAE	1	0
Number of subjects who discontinued due to TEAE	0	0
Number of subjects who died	0	0

**Table 5:** Adverse events related to the investigational drug after administration of BS‐50a or bevacizumab (AEs observed more than 5% in one of treatment group)

	BS‐503a	Bevacizumab
Nasopharyngitis	2	4
Headache	5	8
Somnolence	0	3
Epistaxis	4	1
Rhinorrhea	3	0

The administered dose of 3 mg/kg is lower than the approved clinical dose range of 5–15 mg/kg. Previous PK studies with bevacizumab have established that bevacizumab PK is linear at doses ranging from 1–10 mg/kg, and therefore the chosen dose for this study is within that linear range allowing for PK similarity assessment of BS‐503a (Cobleigh et al. 2003). Regarding evaluation of safety and tolerability, the chosen dose might not be sufficient to estimate safety profile at therapeutic dose. However, no notable difference was observed between BS‐503a and bevacizumab, and no new findings were observed compared to the previous knowledge of bevacizumab. Thus, this provides confidence that no critical difference between BS‐503a and bevacizumab may be observed on the safety profile at therapeutic dose levels.

In this study, an SAE of duodenal ulcer perforation was observed 47 days after administration of BS‐503a. Considering the long half‐life of bevacizumab, this SAE may be related to the study drug. Gastrointestinal perforations are reported for bevacizumab as rare (0.3–2.4%) but severe, sometimes fatal, outcome in the bevacizumab Patient Insert Leaflet (PIL) (Food and Drug Administration, 2013). Intra‐abdominal inflammatory process may be a risk factor for gastrointestinal perforations in patients with metastatic carcinoma of the colon or rectum (EMA, 2014). The subject had no history of gastric or duodenal ulcers, or no history of recent surgery, but was infected with *Helicobacter Pylori*, which could be a risk factor for gastrointestinal ulcers and perforations (Marshall and Warren 1984). It may be worth screening subjects for *H. Pylori* infection prior to inclusion into a healthy volunteer study with bevacizumab (and biosimilar candidates) to exclude high‐risk subjects.

No notable differences were observed on ADA formation between two groups; however, incidence was much higher than reported values (0.63%) (Food and Drug Administration, 2013). A potential reason for the ADA read‐out to be so different to that observed may be due to high levels of soluble target in blood which could causes false‐positive results (Dai et al. 2014). In the validation of the bioanalytical method, to detect ADA, specificity was confirmed using drug‐added serum. However, after administrating bevacizumab, VEGF homodimer could be formed and caused false‐positive results. Further investigation will be necessary for immunogenicity assessment in future studies.

In conclusion, in this study, BS‐503a, a proposed biosimilar, is shown to have highly similar PK parameters to the originator product and as a result can undergo further clinical assessment.

## Author Contributions

N. Tajima designed the study and wrote manuscript. A. Martinez managed the study and involved in revision of the manuscript. F. Kobayashi designed statistics involved in revision of the manuscript. L. He led bioanalysis and developed the bioanalytical method and conducted the sample assay. P. Dewland is a medical advisor and contributed in revision of the manuscript.

## Disclosures

All authors are employees of Daiichi Sankyo Co., Ltd or its affiliates.

## Ethical Approval

All procedures performed in studies involving human participants were in accordance with the ethical standards of the institutional and/or national research committee and with the 1964 Helsinki declaration and its later amendments or comparable ethical standards.
